# Single‐cell analysis reveals innate immunity dynamics in ankylosing spondylitis

**DOI:** 10.1002/ctm2.369

**Published:** 2021-03-21

**Authors:** Jing Liu, Yulong Tang, Yan Huang, Jian Gao, Shuai Jiang, Qingmei Liu, Yanyun Ma, Xiaolin Qian, Feng Qian, John D. Reveille, Dongyi He, Hejian Zou, Li Jin, Qi Zhu, Weilin Pu, Jiucun Wang

**Affiliations:** ^1^ State Key Laboratory of Genetic Engineering, Collaborative Innovation Center for Genetics and Development, School of Life Sciences, and Human Phenome Institute Fudan University Shanghai China; ^2^ Division of Dermatology, Huashan Hospital Fudan University Shanghai China; ^3^ Ministry of Education Key Laboratory of Contemporary Anthropology, Department of Anthropology and Human Genetics, School of Life Sciences Fudan University Shanghai China; ^4^ Institute for Six‐Sector Economy Fudan University Shanghai China; ^5^ Division of Rheumatology and Clinical Immunogenetics, McGovern Medical School The University of Texas Health Science Center Houston Texas USA; ^6^ Institute of Arthritis Research, Shanghai Academy of Chinese Medical Sciences Guanghua Integrative Medicine Hospital Shanghai China; ^7^ Division of Rheumatology, Huashan Hospital Fudan University Shanghai China; ^8^ Department of Rheumatology Shanghai Guanghua Hospital of Integrated Traditional Chinese and Western Medicine Shanghai China; ^9^ Institute of Rheumatology, Immunology, and Allergy Fudan University Shanghai China; ^10^ Research Unit of Dissecting the Population Genetics and Developing New Technologies for Treatment and Prevention of Skin Phenotypes and Dermatological Diseases (2019RU058) Chinese Academy of Medical Sciences Shanghai China

Dear editor,

Ankylosing spondylitis (AS) is a hereditary and immune‐mediated disease with diverse clinical manifestations, including chronic back pain and spine stiffness.^1^, ^2^, ^3^ Till now, the exact etiology of AS remains largely unknown. Many studies considered AS to be an autoimmune disease and focused on the adaptive immune responses in AS.^4^ Nevertheless, recent studies have classified AS as an auto‐inflammatory disease, suggesting that innate immune abnormalities may play a crucial role in AS pathogenesis.^5^ However, the characterizations of innate immune cells, especially the myeloid cells and natural killer (NK) cells, are significantly underexplored in AS.^6^, ^7^ Therefore, we performed the first single‐cell sequencing study of AS focusing on monocytes and NK cells.

To comprehensively characterize the transcriptomes of AS patients between different disease stages and medication status, we recruited four early‐stage AS patients before and after treatment with etanercept (a kind of tumor necrosis factor‐alpha [TNF‐α] blockers, which is widely used for AS treatment^8^, ^9^), two late‐stage AS patients, and two healthy donors (Figures 1A and S1A; Table S1). Monocytes and NK cells were isolated from peripheral blood mononuclear cells using magnetic beads with high specificity (Supporting Information). After stringent quality control procedures, about 70,000 monocytes and NK cells (Table S2) were retained for further analysis (Figures 1B, 1C, and S1B). We identified three monocyte subgroups based on canonical marker genes and literature searching, including MS1 characterized by *S100A8* and *VCAN*; MS2 defined by *CDKN1C* and *FCGR3A*; and MS3 characterized by *CD1C*. Similarly, we defined five distinct NK cell states by *FCGR3A* and *FCER1G* (NS1); *FCGR3A* and lower expression of *FCER1G* (NS2); *HLA‐DRB1* (NS3); decreased expression of both *FCGR3A* and *NCAM1* (NS4); and lower expression of *FCGR3A*, and higher expression of *NCAM1* (NS5) (Figures 1D, S1C, and S1D).

**FIGURE 1:**
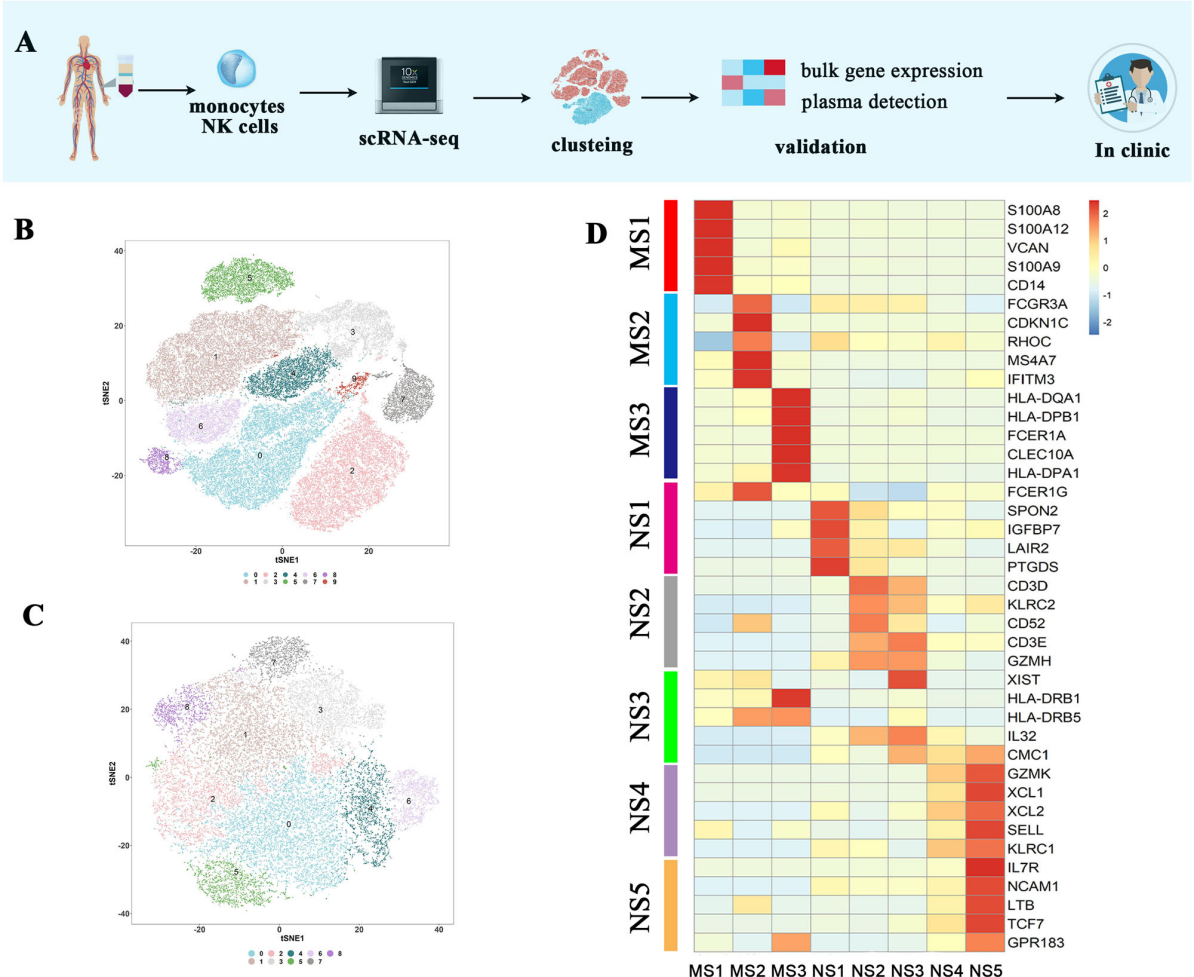
Donor characteristics and analysis strategy. (A) Processing pipeline of blood samples from AS patients and healthy controls. Monocytes and NK cells were separated from other peripheral blood mononuclear cells (PBMCs) by antibody‐coated beads, and subsequently loaded onto the 10× scRNA‐seq platform. (B and C) tSNE plot for monocytes (*n* = 49.8 × 10^3^) and NK cells (*n* = 26.2 × 10^3^) showing different clusters. (D) Heatmap of selected markers revealed the differential expression profile of each cell state (false discovery rate [FDR] < 0.05)

After cell subclustering, we focused on the transcriptional changes of monocytes and performed differential expression (DE) analysis between early‐stage AS patients without TNF‐α blocker treatment and healthy controls within the MS1 population, which occupied the majority of monocytes (Figures 2A and S2A). Surprisingly, few differential expressed genes (DEGs) were found, indicating low inflammation activation in MS1 cells of AS patients. However, inflammatory scores composed by multiple key chemokines (Supporting Information and Figure 2B), as well as several crucial inflammatory pathways, including nuclear factor‐kappa B, interleukin‐17 (IL‐17), TNF, and Toll‐like receptor (TLR) pathways, were significantly downregulated after etanercept treatment (Figures S2B and S2C). Moreover, expression of those chemokines also decreased in plasma after etanercept treatment (Figure 2C). Importantly, it suggested that inflammation levels in MS1 were lower than those previous thought, and TNF‐α blocker treatment might primarily target monocytes through inhibiting the above crucial inflammatory pathways.

**FIGURE 2:**
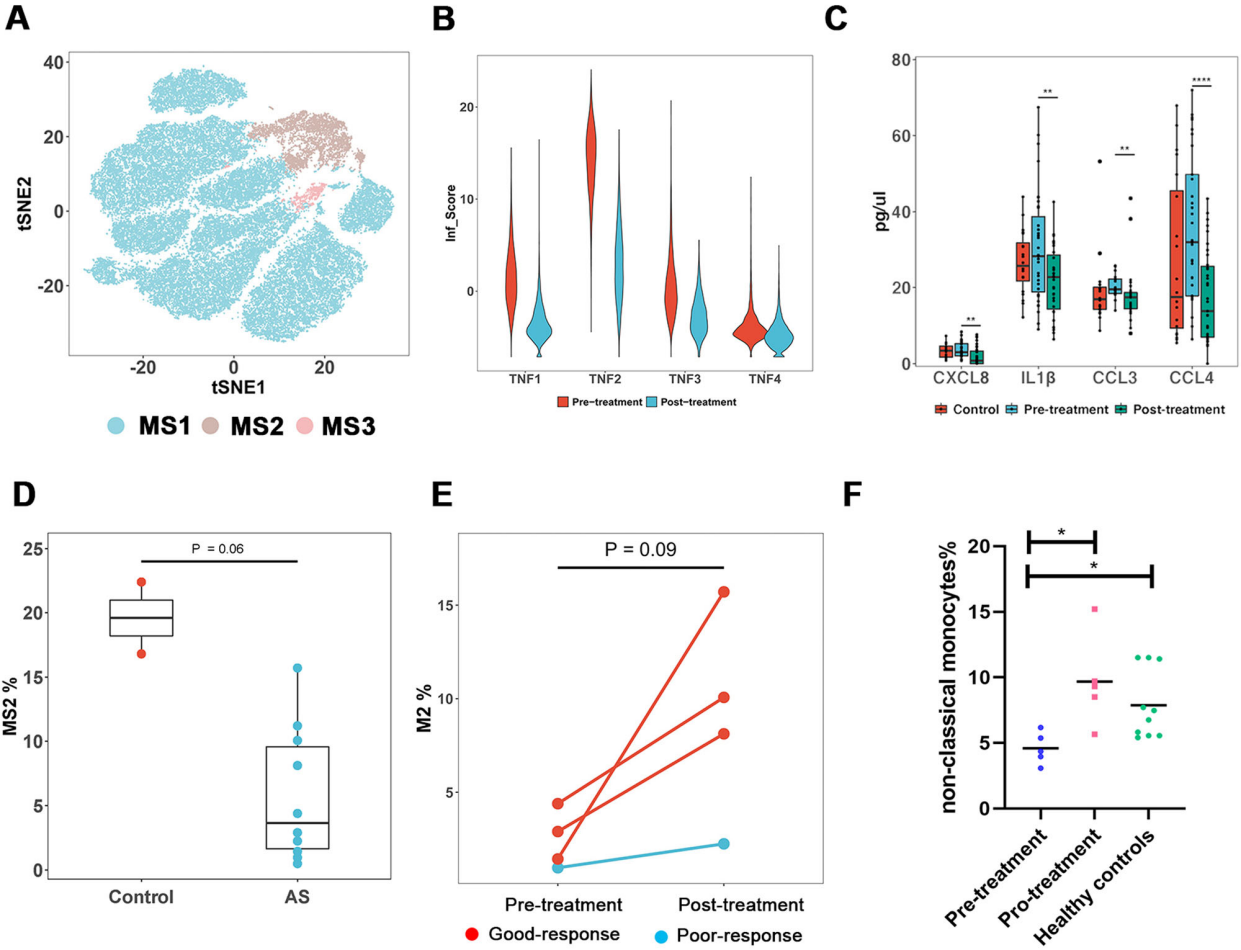
Characteristics of monocytes in AS patients. (A) tSNE plot showing three monocyte states (MS1, MS2, and MS3). (B) Inflammatory score (Inf_Score) defined by the expression of eight genes (CCL4L2, CCL4, CXCL8, CCL3L1, IL1B, CCL3, FOS, and TNF) of four AS patients treated with etanercept. The Inf_Score decreased drastically after treatment. (C) Detection of plasma cytokines in controls and patients pre‐ and posttreatment with our four‐cytokine panel including CCL3/CCL4, CXCL8, and IL1β. (D) Comparison of the relative proportion of MS2 cells between AS and healthy controls. (E) Variation of the relative proportion of MS2 after treatment with etanercept. The red pots presented patients with good response to etanercept, whereas blue ones presented patients with poor response. (F) Validation of monocyte signatures associated with TNF blockade by flow cytometry

Next, we investigated the distribution of three monocyte subgroups for each sample. Strikingly, the proportions of MS2 cells against all monocytes were significantly higher in controls than in patients (20% vs. 3%; Figure 2D). Furthermore, the proportions of MS2 cells were associated with the response to etanercept. After 1‐month treatment with etanercept, the percentages of MS2 cells significantly increased from 3%–5% to 10%–15% for the good responders, but remained unchanged for poor responders (Figure 2E). To validate it, we quantified the percentages of MS2 cells in an independent sample group, consisting of five AS patients with good response to etanercept and 10 controls by flow cytometry. In accordance, the percentages of MS2 cells (characterized by CD14^low^CD16^high^) were significantly fewer in AS patients than in controls and were significantly recovered after 1‐month etanercept treatment (Figures 2F and S2D). Taken together, the percentages of MS2 cells were decreased in AS patients and might be recovered by TNF‐α blockers treatment in the good responders but not the poor responders, suggesting that MS2 cell proportion could be a potential indicator to the response to TNF‐α blocker treatment and MS2 cells might be beneficial for AS treatment.

Regarding NK cells (Figure 3A), we found NK cells from healthy controls were largely enriched in the NS1 subgroup, whereas cells in the NS2 subgroup were mainly originated from AS patients, showing significant cell type preferences between AS patients and controls (Figure 3B). Notably, the proportion of NS2 cells (FCER1G^−^FCGR3A^+^ NK cells, similar to memory‐like NK cells) differed significantly between AS patients and healthy controls and might serve as a potential indicator for AS (Figure 3C). Furthermore, we performed deconvolution analysis of bulk NK cell transcriptomes and flow cytometry analysis (Figures 3D–F and S3A). In accordance, all these results confirmed the expansion of FCER1G^−^FCGR3A^+^ NK cells in AS patients, regardless of the TNF‐α blocker treatment. Besides, we conducted DE and pathway enrichment analysis for NS4 and NS5 cells and found that they were pathogenically activated by oxidative phosphorylation, IL‐17, IFN‐γ, and TNF signaling pathways (Figures 3G and S3B). Meanwhile, trajectory analysis suggested that several key transcription genes, including *DUSP1*, *JUN*, and *FOS*, in NS4 and NS5 might contribute to the deviation of these cells in AS patients from healthy controls (Figures 3H, 3I, S3C, and S3D). It is also noteworthy that the abnormal activation of NS4 and NS5 cells could not be reversed by TNF‐α blockers treatment. Overall, we found the expansion of NS2 cells and two pathogenically activated NK cell subgroups in AS patients. The fact that these abnormalities could not be restored by TNF‐α blocker treatment suggested the limitation of TNF‐α blockers in AS treatment, and the roles of these cell subgroups in AS pathogenesis required further exploration.

**FIGURE 3:**
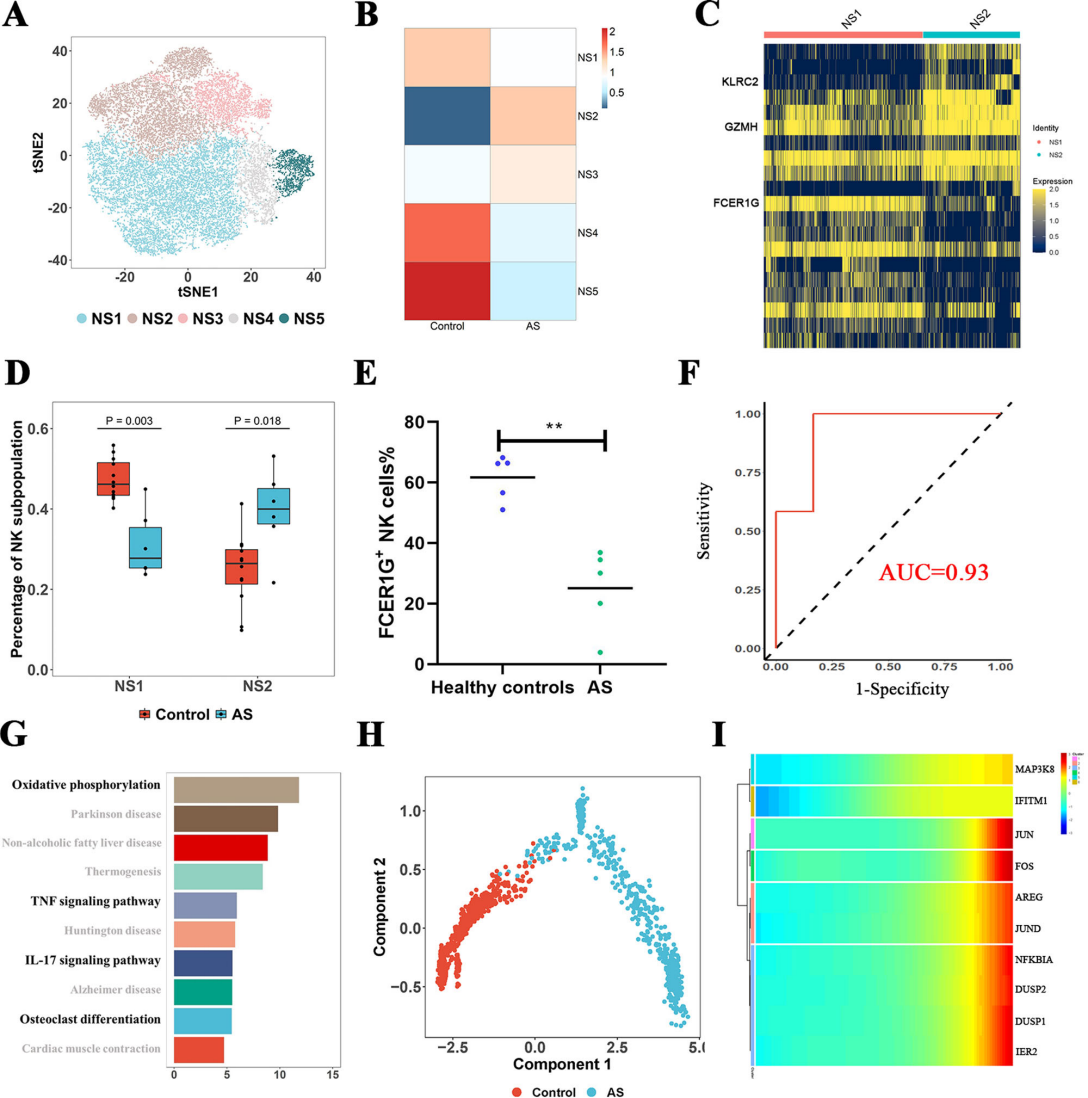
Characteristics of NK cells in AS patients. (A) tSNE plot showing five NK cell states (NS1, NS2, NS3, NS4, and NS5). (B) Scaled distribution of subgroups of NK cells in AS patients and healthy controls. (C) Heatmap plot showing gene expression in NS1 and NS2 subsets. Three genes including *KLRC2*, *GZMH*, and *FCER1G* showed high difference between NS1 and NS2. (D) Validation of NS1 and NS2 proportions in AS patients and healthy controls by deconvolution analysis after bulk RNA sequencing. (E) Flow cytometry staining with CD56, and FCER1G in AS patients and controls. (F) Receiver operating characteristic (ROC) curve of NS1 as discriminator of AS patients and healthy controls. (G) Pathway enrichment analysis (KEGG) of genes differentially expressed between AS patients and controls. Pathways highlighted with bold fonts played an important role in the pathogenesis of AS. (H) Trajectory analysis of NS4. (I) Key genes associated with the pseudo‐time of NS4. Data of NS5 are shown in Supporting Information figures

In conclusion, we profiled the transcriptional landscape of monocytes and NK cells, which are two pivotal cell types of innate immunity in AS at single‐cell resolution. We found that monocytes might be the main target cells of TNF‐α blocker, and the CD14^low^CD16^high^ monocytes, as well as their related inflammatory cytokines, may predict the response to TNF‐α blocker treatment for AS patients. Moreover, we discovered that the memory‐like and CD16^medium/low^ NK cells might be involved in the pathogenesis of AS regardless of TNF‐α blocker treatment, and further functional experiments are required to reveal their roles in AS pathogenesis Figure 4. Our analysis highlights the importance of innate immune cells in AS pathogenesis and may open new possibilities for the therapeutic strategies of AS in the future.

**FIGURE 4:**
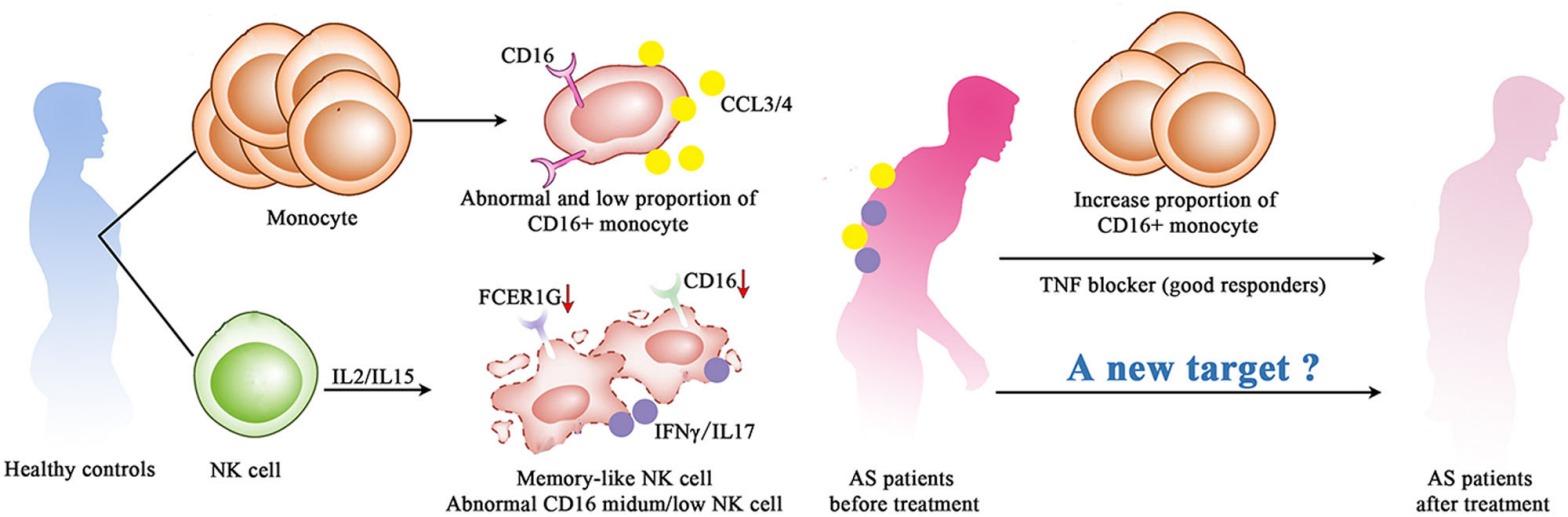
Possible mechanisms involving innate immunity in AS pathogenesis and responses to etanercept. Among monocytes, we identified MS2 (CD16^+^ monocytes, similar to nonclassical monocytes) as a peripheral blood mononuclear cell (PBMC) population present at lower proportion in AS patients. Treatment with etanercept recovered a higher proportion of MS2 in good responders. Among NK cells, a subtype similar to memory NK cells was induced and expanded in AS. In addition, CD16^medium/low^ NK cells were pathogenically activated in AS and did not respond to etanercept in any individual

## AUTHOR CONTRIBUTION

Jing Liu and Weilin Pu analyzed the data and drafted the manuscript. Yulong Tang, Yan Huang, Jian Gao, Shuai Jiang, and Xiaolin Qian performed the experiment. Qingmei Liu, Feng Qian, John Reveille, Hejian Zou, Dongyi He, and Li Jin revised the manuscript. Yanyun Ma recruited the participants. Qi Zhu and Jiucun Wang designed the project and revised the manuscript. All authors approved the final version of the article, including the authorship list.

## DATA AVAILABILITY STATEMENT

The data that support the findings of this study are available from the corresponding author upon reasonable request.

## CONFLICT OF INTEREST

The authors declare no conflict of interest.

## Supporting information

Supporting Information

Figure S1

Figure S2

Figure S3

Figure S4
