# Supplementary material for: Single‐cell analysis reveals innate immunity dynamics in ankylosing spondylitis
Source: Clin Transl Med. 2021 Mar 21;11(3):e369. doi: 10.1002/ctm2.369 (PMC7982614; doi:10.1002/ctm2.369)
Supplement: Supplementary file 1 — Supporting Information [file CTM2-11-e369-s003.docx]

**Methods**

**Study participants**

Twelve samples of PBMCs, including eight from four AS patients treated with etanercept (pre/post treatment), two from late-stage AS, and two from healthy controls were recruited (Table S1). All the participants signed written informed consents. This study was approved by the Ethical Committees of the School of Life Sciences of Fudan University. All patients fulfilled the 1984 modified New York criteria for AS. Clinical data were collected including those correlating with disease activity (Bath Ankylosing Spondylitis Disease Activity Index [BASDAI], C-reactive protein [CRP], erythrocyte sedimentation rate [ESR], Ankylosing Spondylitis Disease Activity Score [ASDAS]), and structural damage (modified Stoke Ankylosing Spondylitis Spinal Score [mSASSS]). We performed validation analysis on additional healthy controls and pre/post-treated AS patients (Table S3). In addition, good responders to etanercept were defined as ΔASDAS>1.1, while ASDAS of the poor responder deteriorate after treatment.

This study proceeded along different stages that include independent samples as following.

1. Discovery stage: single-cell sequencing

We recruited 12 donors, including two healthy controls, four AS patients naïve for TNF blocker, and two late-stage AS patients, both of affected by ankylosis. We collected the blood of the four patients naïve for TNF blockers before (baseline) and after 1-month treatment with etanercept.

1. Bulk RNA sequencing

To further validate the results related to NK cells, we recruited another cohort of six AS patients who were naïve for TNF blocker, and 12 healthy controls. We separated NK cells with beads (Miltenyi Biotec) and performed RNA sequencing.

1. Flow cytometric analysis

To further validate the result related to monocytes, we recruited an independent cohort of five AS patients also naïve for TNF blockers. We collected blood samples at baseline and after 2-weeks treatment with etanercept.

1. Cytokine detection

For this phase, we collected further 35 AS patients naïve for TNF blocker, and 20 healthy controls. We collected the plasma at baseline and after 3-month treatment with etanercept.

**Pipelines of single-cell RNA sequencing (scRNA-seq)**

Monocytes and NK cells were separated from other PBMCs by beads (Miltenyi Biotec, Monocyte: #130-050-201, NK cells: #130-092-657). We also performed flow cytometry to validate the specificity of above cell types (**Figure S4**). Next, monocytes and NK cell numbers were determined, and cells were mixed at 1:1 ratio. Then, Single-cell RNA sequencing (scRNA-seq) of the samples was performed with 10x Genomics, according to the manufacturer’s recommendations. Data analysis involved several steps, including quality control and batch effect correction.

**Single-cell RNA sequencing and reads processing**

Cell suspension from each blood sample was subjected to 3’ single-cell RNA sequencing using the Gel Bead Kit V3 (10x Genomics, Pleasanton, CA, USA). After sequencing, Cell Ranger toolkit (v3.1) was used to align the droplet-based sequencing data against GRCh38 human reference genome. Seurat (v3.1.4) was then applied to the data for downstream analysis.

**Quality control and batch effect correction of scRNA-seq data**

We first discarded cells with low quality, based on two criteria: contamination rate with mitochondrial genes (<20%) and lower and upper limits of unique molecular identifiers in the range of 500 < UMIs < 5000. Moreover, to remove candidate doublets, we applied the DoubletFinder package with the default settings [1]. The quality control and normalization procedures of the scRNA-seq data were conducted with Seurat (v3.1.4) [2]. Specifically, we first employed a global-scaling normalization method “LogNormalize”, which normalizes the feature expression measurements for each cell by the total expression, multiplies the result by a scale factor (10,000), and log-transforms the result using the NormalizeData() function in Seurat. Next, we merged the scRNA-seq data of each sample using the merge() function in R v3.6.3. To correct the batch effects, we conducted the matching mutual nearest neighbors (MNNs) correction [3], and used its implementation in Python (mnnpy, https://github.com/chriscainx/mnnpy), which is a faster version than that implemented in R. We used the FindVariableFeatures() function to obtain the top 5000 highly variable genes (HVGs) of all samples, and as the input dataset for batch effect correction. Finally, we used the batch effect-corrected and scaled gene expression data of all samples for downstream analysis.

**Unsupervised dimensional reduction and clustering**

The batch effect-corrected gene expression profiles of the top 5000 HVGs were then used to compute the principal components (PCs). The PCElbowPlot() function of Seurat was then utilized to select the optimal number of PCs for further analysis. FindNeighbors() and FindClusters() functions in Seurat were used for cell clustering. To find the optimal cluster resolution, a visualization-based method “clustering tree” was applied when required [4]. Moreover, RunTSNE() function was performed for visualization. The cell identities of each cluster were defined through the identification of differential expression of known markers. In the first round of clustering, we identified monocytes (*CD14*, *FCGR3A*, *LYZ*, *CST3*), NK cells (*NKG7*, *KLRB1*, *GNLY*), T cells (*CD3D*, *CD8A*, *IL7R*), B cells (*IGKC*, *JCHAIN*), eosinophils (*CLC*), neutrophils (*FCGR3B*), hematopoietic stem cells (*CD34*, *GATA2*), contaminating platelet cells (*PPBP*, *PF4*), and few doublets that were not removed by the previous quality control procedures (higher expression of a mixture of multiple known lineage markers). We then excluded all cells that were not monocytes or NK, and conducted a second round of clustering for monocytes or NK cells independently. The procedures for the second round of clustering were the same as for the first, starting from computing the PCs, then conducting the cell clustering, and determining the cell identity corresponding to each cluster.

**Identification of signature genes of the cell clusters**

We run the Seurat FindAllMarkers() function to identify the genes specifically expressed in each cluster. The significance of the differences in gene expression was determined using the Wilcoxon rank sum test with Bonferroni correction. The signature genes of each cluster were determined based on following criteria: 1) expressed in more than 20% of the cells within either or both two groups; 2) |log_2_FC| > 0.5; 3) Wilcoxon rank sum t test adjusted p-value < 0.01.

**Inference of cell differentiation state using trajectory analysis**

The trajectory analysis was performed using the Monocle2 package (v2.14.0) to reveal cell state transitions. We used the top 100 differentially expressed genes (DEGs) of each subgroups identified through Seurat in MS2, NS4, and NS5 cells, to sort the cells in pseudo-time order. Meanwhile, the DDRTree() function in Monocle2 was applied to reduce the dimensions while plot_cell_trajectory() was run for visualization. The DEG changes along the pseudo-time were also determined with the differentialGeneTest() function of Monocle2, and visualized with the function plot_pseudotime_heatmap().

**Inflammatory score**

Next, we designed a panel assay generating the expression profile of more than eight inflammatory genes related to NFκB and IL17 pathways, which is referred to as Inf_Score. We found that the Inf_Score decreased after one-month treatment with etanercept. Eight genes included CCL4L2, CCL4, CXCL8, CCL3L1, IL1B, CCL3, FOS, and TNF.

**Bulk-RNA sequencing process**

The sequencing quality of the bulk RNA-seq datasets of NK cells and monocytes from peripheral blood was assessed with FastQC. After quality control, the data were quantified by kallisto (v0.46.1) against the annotated transcripts (Gencode v24). Gene expression level was calculated by aggregating transcript expression (TPM) belonging to the same gene with tximport.

**Deconvolution of bulk-RNA sequencing**

We performed the scRNA-seq-based deconvolution analysis using the BisqueRNA package [5]. Our scRNA-seq dataset, including NK and monocytes, was used as reference to deconvolute the cell type percentage from the bulk RNA-seq data. Specifically, we deconvoluted the bulk RNA-seq data of NK and monocytes respectively into five (NS1-NS5) and three (MS1-MS5) subtypes according to the scRNA-seq data. The differences in cell type percentage between different groups were assessed by Wilcoxon rank sum test (AS vs. control; post- vs. pre-treatment).

**Memory-like NK cell staining**

Prior to staining, FcεRIγ (Merck Millipore) were conjugation with iFluor™ 488 fluorescent used a ReadiLink™ Rapid iFluor™ 488 Antibody Labeling Kit (AAT Bioquest).For staining protocol, antibodies to the following surface molecules were used for cell staining: CD3 (UCHT1) (from BioLegend) and CD56 (NCAM16.2), CD45 (HI30) (all from BD Biosciences). 150 μl of anticoagulated peripheral whole blood was lysed with 1x BD FACS™ lysing solution (BD Biosciences) for 10 minutes at room temperature, then stained with surface antibodies for 15 minutes at room temperature for 15 minutes in the dark. The samples were centrifuged and washed twice at 300g for 5 minutes (PBS, and PBS containing 1% FCS). Intracellular staining for FcεRIγ was used Fixation/Permeabilization Solution Kit (BD Biosciences). All samples were acquired on a CytoFLEX LX cytometer (Beckman Coulter) and analyzed using FlowJo software version 10.7.1 (BD Biosciences) (**Figure S3A**).

**Figure S1 Identification of cell differentiation states** (A) Numbers of monocytes and NK in each sample. (B) tSNE plot with low resolution. Identification of monocyte and NK cells based on gene signatures. (C) Violin plot showing the signatures of different monocyte states. CD14^high^ cells correspond to MS1, CD14^low^CD16^high^ cells to MS2, and high expression of *CD1C* and *FCER1A* define MS3. (D) Violin plot showing the signatures corresponding to different NK cell states. NS1 was defined by high expression of *FCGR3A, FGFBP2*, and *FCER1G*; NS2 by high expression of *FCGR3A* and *FGFBP2*, and low expression of *FCER1G*; NS3 by high expression of *MHCII* molecules; NS4 by intermediate expression of *FCGR3A* and low expression of *NCAM1*; NS5 by high expression of *NCAM1* and low expression of *FCGR3A*.

**Figure S2** (A) Sample distribution among the clusters defined in monocytes (B) Pathway enrichment analysis comparison with AS before treatment and after treatment. (C) Variation of prominent genes’ expression including CCL4L2, CCL4, CXCL8, CCL3L1, IL1B, CCL3, FOS, and TNF in AS patients pre- and post-treatment. (D) Gating strategy to identify MS2 by flow cytometry.

**Figure S3** (A) Gating strategy to identify NS1 and NS2 by flow cytometry. Whole blood was first stained with CD45 to determine PBMCs, which were then stained with CD3 and CD56 to determine NK cells. Last, it was stained with FCER1G to determine memory-like NK cell. (B) Pathway enrichment analysis (KEGG) of genes differentially expressed between AS patients and controls in NS5. (C) Trajectory analysis of NS5. (D) Key genes associated with the pseudo-time of NS5.

**Figure S4** CD14 staining for isolated monocytes and CD56 staining for isolated NK cells. Top : CD14 staining for PBMC (left), and beads enriched cells (right). Bottom: CD56 and CD3 for isolating NK cells. Left: PBMC before separation. Right: cell separation after enriching by beads.

**Table S1 Clinical characteristics of individuals included for scRNA-seq**

| sample number | treatment | age | HLA-B27 | BASDAI | BASFI | CRP(mg/L) | ESR(mm/h) | ASDAS-CRP |
| --- | --- | --- | --- | --- | --- | --- | --- | --- |
| C1 |  | 39±18.4 |  |  |  |  |  |  |
| C2 |  |  |  |  |  |  |  |  |
| TNF1_pre | naïve | 37.5±10.5 | positive | 4.7 | 1.7 | 86.60 | 17 | 3.75 |
| TNF1_pro | 1 month |  | positive | 4.6 | 1.6 | 70.48 | 40 | 3.98 |
| TNF2_pre | naïve |  | positive | 3.0 | 4.9 | 33.81 | 56 | 3.25 |
| TNF2_pro | 1 month |  | positive | 1.9 | 1.0 | 10.74 | 10 | 2.08 |
| TNF3_pre | naïve |  | positive | 2.4 | 1.4 | 36.90 | 29 | 2.90 |
| TNF3_pro | 1 month |  | positive | 0.5 | 0.0 | 1.07 | 2 | 1.04 |
| TNF4_pre | naïve |  | positive | 6.6 | 2.1 | 34.10 | 48 | 4.45 |
| TNF4_pro | 1 month |  | positive | 3.3 | 0.5 | 7.40 | 12 | 2.98 |
| Ankylosis1 | naïve |  | positive | 3.5 | 9.4 | 18.36 | 18 | 3.31 |
| Ankylosis2 | 5 year |  | positive | 2.5 | 6.4 | 1.64 | 3 | 1.45 |

**Table S2 Distribution of cell numbers in each individual**

|  | AS1 | AS2 | N1 | N2 | TNF1_0 | TNF1_2 | TNF2_0 | TNF2_2 | TNF3_0 | TNF3_2 | TNF4_0 | TNF4_2 |
| --- | --- | --- | --- | --- | --- | --- | --- | --- | --- | --- | --- | --- |
| MS1 | 203 | 3511 | 1972 | 5112 | 3111 | 7699 | 3719 | 3719 | 3687 | 2817 | 3998 | 5391 |
| MS2 | 1 | 459 | 575 | 1056 | 31 | 179 | 55 | 694 | 113 | 250 | 184 | 606 |
| MS3 | 1 | 126 | 20 | 116 | 69 | 96 | 56 | 5 | 95 | 11 | 9 | 21 |
| NS1 | 20 | 1968 | 1072 | 3422 | 906 | 473 | 265 | 1221 | 1480 | 675 | 190 | 494 |
| NS2 | 37 | 1240 | 90 | 123 | 303 | 196 | 355 | 1291 | 2198 | 1259 | 116 | 241 |
| NS3 | 13 | 247 | 672 | 14 | 64 | 35 | 86 | 245 | 206 | 117 | 506 | 778 |
| NS4 | 0 | 229 | 86 | 916 | 133 | 89 | 87 | 91 | 186 | 128 | 44 | 99 |
| NS5 | 2 | 75 | 40 | 797 | 85 | 66 | 94 | 80 | 51 | 46 | 71 | 54 |

| Bulk RNA-sequencing for NK cells | | | Cytokine detection | | | Flow cytometry | | |
| --- | --- | --- | --- | --- | --- | --- | --- | --- |
|  | AS | HS | Pre | Post | HS | Pre | Post | HS |
| numbers | 6 | 12 | 35 | 35 | 20 | 5 | 5 | 10 |
| age | 34.83±8.31 | 33.4±4.04 | 34.32±9.92 | | 34.26±7.01 | 46.2±10 | | 44.1±10.1 |
| BASDAI | 5.53±1.10 |  | 5.24±0.86 | 1.39±0.88 |  | 4.65±1.70 | 2.48±0.84 |  |
| ASDAS | 3.93±0.77 |  | 3.23±0.75 | 1.02±0.59 |  | 3.52±0.84 | 1.51±0.57 |  |
| CRP | 42.6±25.37 |  | 18.04±18.35 | 2.67±3.54 |  | 25.05±12.73 | 1.90±1.89 |  |
| ESR | 18.6±9.84 |  | 21.42±21.36 | 5.26±4.90 |  | 42±21.33 | 15.80±11.92 |  |
| TNF blocker | naïve |  | naïve | 3-month treatment |  | naïve | 2-week treatment |  |

**Table S3 Clinical characteristics of individuals included for validation**

HS: healthy controls

Pre: AS patients before treatment

Post: AS patients after treatment

**Reference**

[1] McGinnis, C.S., L.M. Murrow, and Z.J. Gartner, DoubletFinder: Doublet Detection in Single-Cell RNA Sequencing Data Using Artificial Nearest Neighbors*.* *Cell Syst*, 2019. 8(4): p. 329-337 e4.

[2] Stuart, T., A. Butler, P. Hoffman, et al., Comprehensive Integration of Single-Cell Data*.* *Cell*, 2019. 177(7): p. 1888-1902 e21.

[3] Haghverdi, L., A.T.L. Lun, M.D. Morgan, et al., Batch effects in single-cell RNA-sequencing data are corrected by matching mutual nearest neighbors*.* *Nat Biotechnol*, 2018. 36(5): p. 421-427.

[4] Zappia, L. and A. Oshlack, Clustering trees: a visualization for evaluating clusterings at multiple resolutions*.* *Gigascience*, 2018. 7(7).
