# Supplementary figures and images for: Single‐cell analysis reveals innate immunity dynamics in ankylosing spondylitis
Source: Clin Transl Med. 2021 Mar 21;11(3):e369. doi: 10.1002/ctm2.369 (PMC7982614; doi:10.1002/ctm2.369)

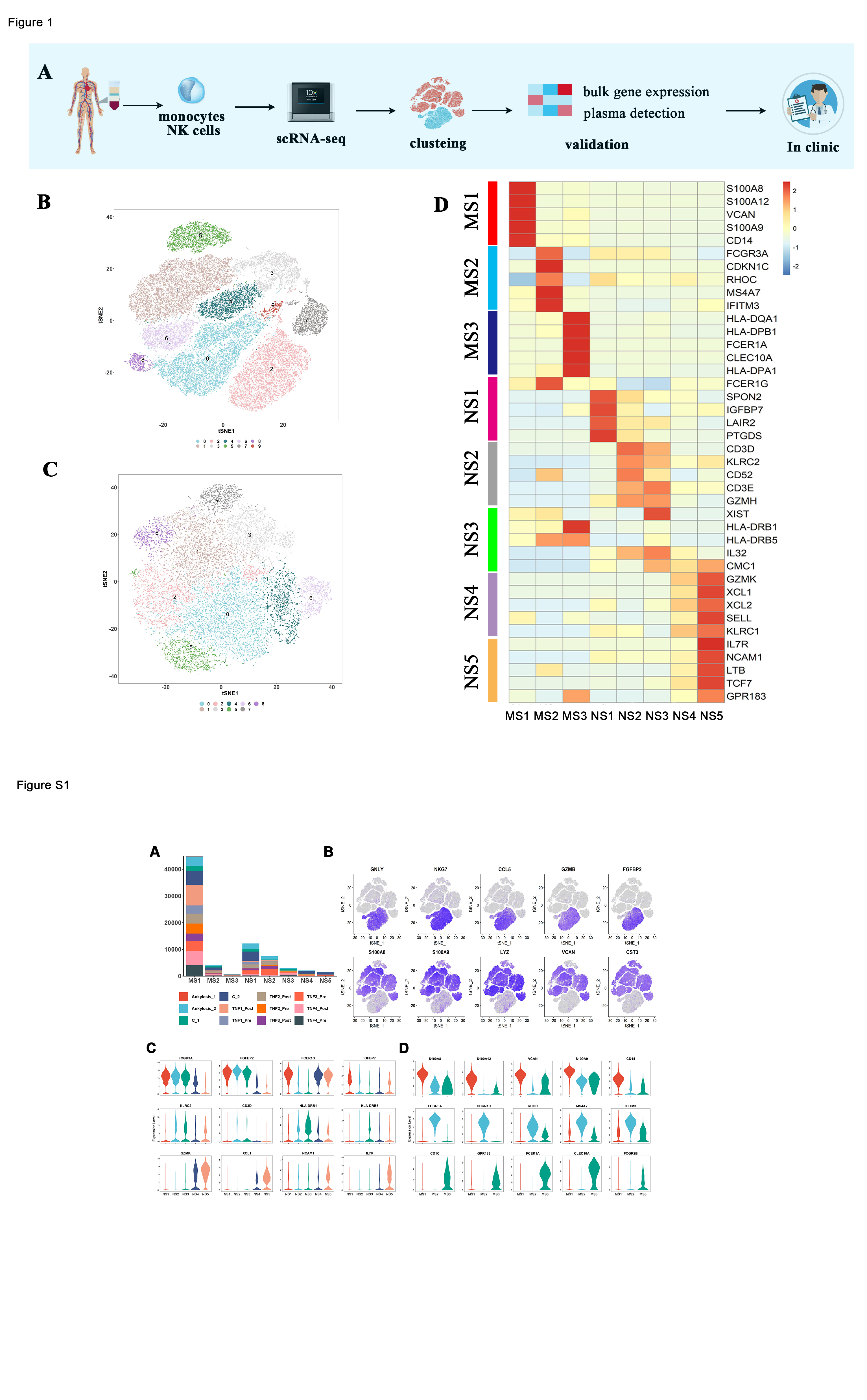

Supplement: Supplementary file 2 — Figure S1 [file CTM2-11-e369-s001.jpg]

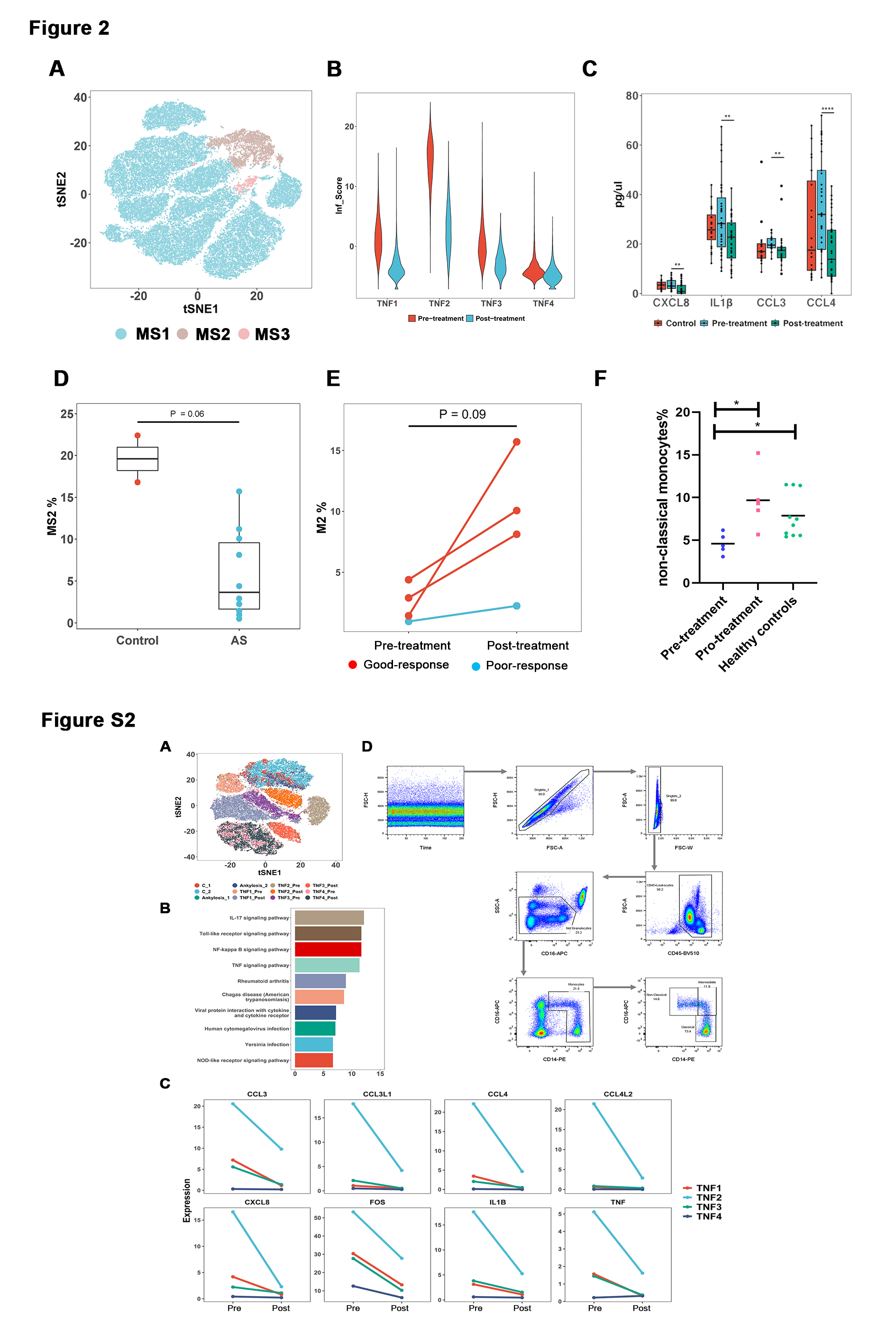

Supplement: Supplementary file 3 — Figure S2 [file CTM2-11-e369-s004.jpg]

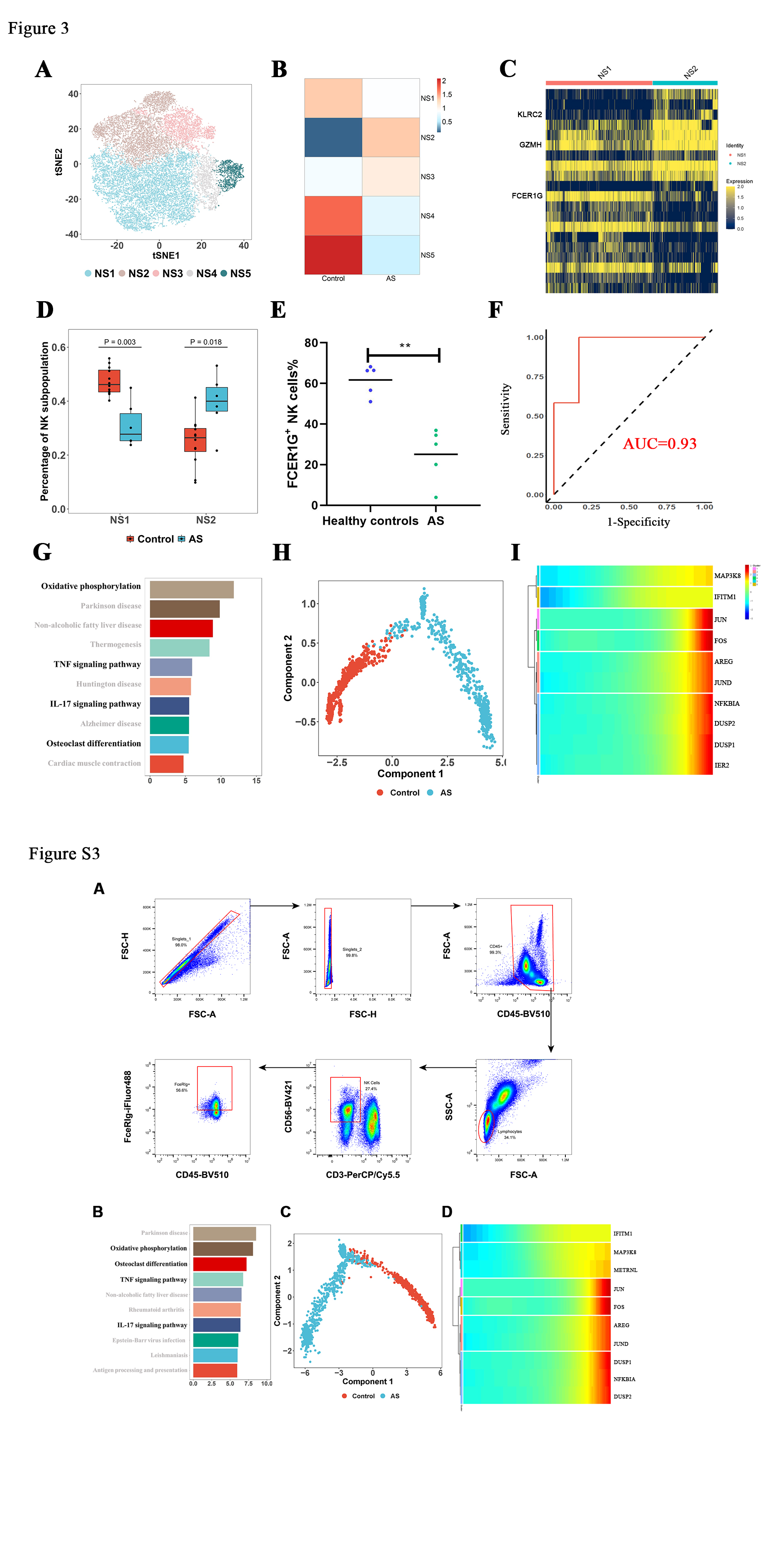

Supplement: Supplementary file 4 — Figure S3 [file CTM2-11-e369-s005.jpg]

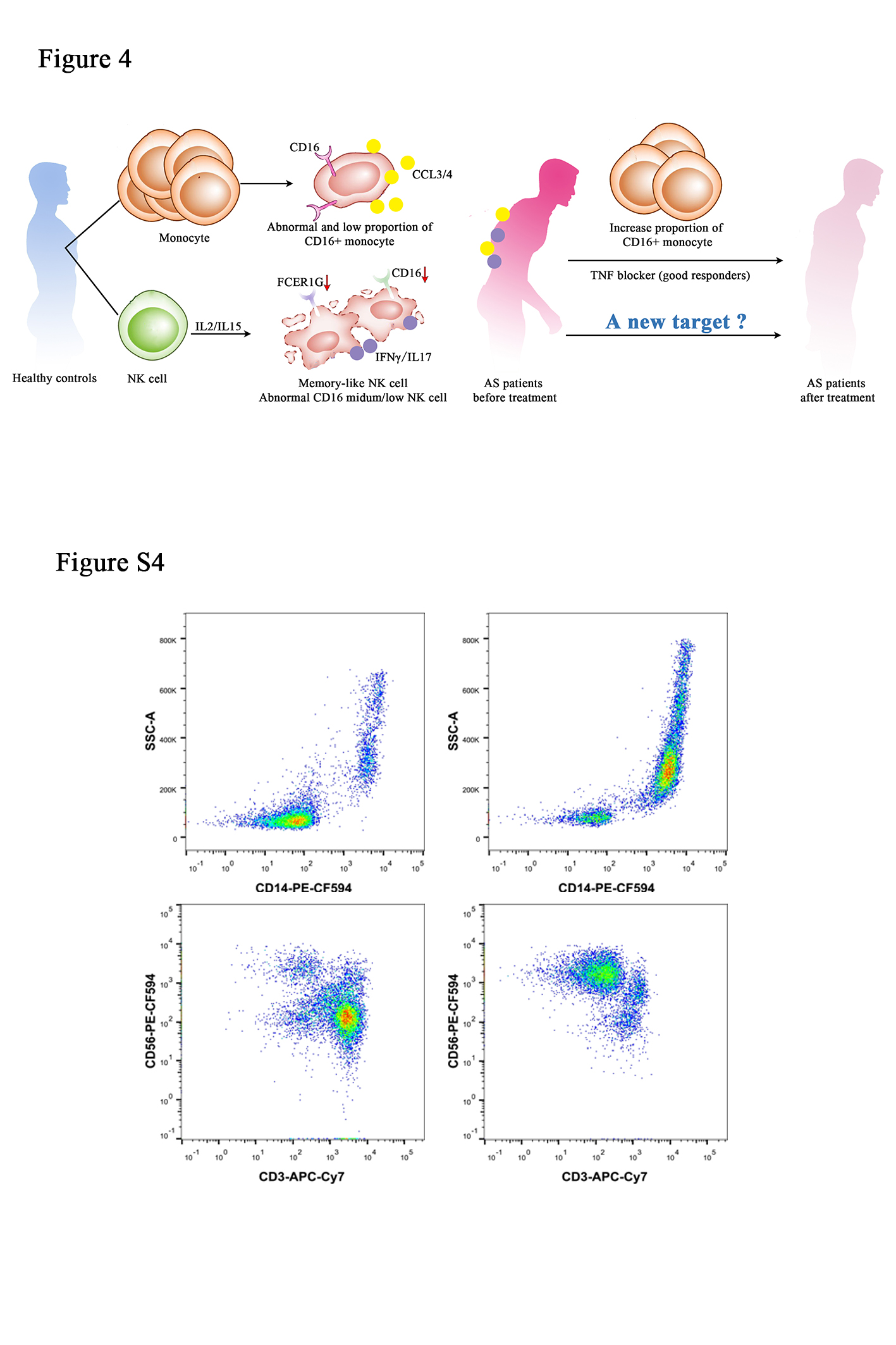

Supplement: Supplementary file 5 — Figure S4 [file CTM2-11-e369-s002.jpg]
